# Cyber warfare: a study of Zelenskyy’s social media political performance strategies and effects

**DOI:** 10.3389/fpsyg.2024.1478639

**Published:** 2024-12-10

**Authors:** Liqiang Wang, Ruonan Wang

**Affiliations:** School of Journalism and Communication, Shandong University, Jinan, China

**Keywords:** Russia-Ukraine war, Zelenskyy, social media, political performance, war narrative frame

## Abstract

During the Russia-Ukraine war, Ukrainian President Volodymyr Zelenskyy has strategically used social media to appeal for international support. This reflects a broader trend of political figures relying on digital platforms to shape public opinion and influence global narratives during crises. This work uses three main analysis methods, content, sentiment and social network analysis. The searched and collected dataset consists 604 valid tweets and 58,100 corresponding comments. The findings show that Zelenskyy employs both textual and visual narratives to construct a war-related agenda, influencing international public discourse. His agenda-setting is most effective in the early stages of the conflict but weakens over time. This study highlights Zelenskyy’s flexibility and adaptability in his media strategy, illustrating the evolving nature of political performance in a globalized media landscape. To maintain effective communication and image-building, leaders must balance audience psychology with the characteristics of digital media.

## 1 Introduction

On February 24, 2022, Russian President Vladimir Putin delivered a televised speech, announcing a “special military operation” in Ukraine aimed at “demilitarization and denazification.” While the world witness brutal clashes on the ground battlefield, the battles in the information and cognitive domains are equally intense. Scholars argue that the Russia-Ukraine war is the first war in which a localized conflict in the physical world has been highly integrated with a globalized information war in cyberspace (Fang and Zhong, 2022). Especially in the present, the immediacy of social media enables interaction and mutual influence between governments and the public worldwide (Fu et al., 2023). Social media platforms have become the main battlefield for competing discourses from all sides.

The war has led to an increase in media audiences in both Ukraine and Russia (Ciuriak, 2022; Laruelle, 2022). Both countries actively use social media for war propaganda, striving to seize the initiative in international discourse. Generally, countries with greater political power generally receive more media attention than those with less power (Dyczok and Chung, 2022). This phenomenon is equally evident in the focus on the performance of political leaders. However, in this cyber warfare, Russia, traditionally seen as a powerful participant in the global information space, has faced significant challenges in its attempts to dominate the international narrative. In contrast, the actor-turned-president of Ukraine, Volodymyr Zelenskyy, has emerged with a new form of political performance, achieving “viral spread” (Specia, 2022), and cohering significant political mobilization value.

Nowadays, social media has become a vital tool for political leaders to communicate politically and shape a strong leadership image (Uali et al., 2023). Zelenskyy’s media performance transformed his pre-war populist political stance into a productive leadership style, demonstrating the immense potential of social media in wartime leadership mobilization. In this context, the study focuses on Zelenskyy’s wartime Twitter account (now rebranded as “X” but still referred to as “Twitter” to avoid confusion), aiming to explore the political performance strategies he employed on social media to construct a war narrative in the background of a prolonged conflict. Additionally, it seeks to understand how Zelenskyy uses these strategies to set relevant agendas within the global discourse system and to examine the effect of these media strategies in terms of agenda-setting outcomes.

Although much has been studied about social media’s role in political communication, most research focuses on its mechanisms in non-war contexts, such as public health crises (Paul and Das, 2023; Terry et al., 2023), elections (Bronstein et al., 2018; Criado et al., 2012; Karlsen, 2015), and foreign policy (Duncombe, 2017). These studies examine social media’s role in information dissemination, political engagement, and policy feedback (Yavetz and Aharony, 2021). However, there is a notable gap in the literature concerning how political leaders utilize social media during wartime to shape international discourse and mobilize support. Few studies have systematically explored this issue, and an overarching theoretical framework is lacking. This study aims to fill this gap by constructing an integrated analytical framework that combines framing theory, dramaturgy theory, and network agenda-setting theory. Through this framework, we seek to conduct an innovative analysis of political leaders’ narratives, performances, and the effects of their social media use in wartime contexts.

Placing the use of social media within the broader context of global networked information warfare, the study examines the decision-making styles and narrative characteristics of leaders from warring parties. This is crucial for accurately understanding the unique role of political leaders in the context of war. By constructing a comprehensive analytical framework, the study aims to clarify the multiple functions of social media in shaping national images, guiding public opinion, and influencing international relations. Not only does this offer new theoretical perspectives and empirical materials for academia, but the findings also hold significant practical implications for policymakers as they adapt to the dynamic shifts in global political discourse and address current and future challenges in international communication.

## 2 Literature review

### 2.1 The mediatization of politics and conflict under cyber warfare

Over the past several decades, advances in technology have transformed communications and the ability to acquire, disseminate, and utilize information in a range of environments (Shonhe and Jain, 2017). With the support of technological empowerment, “cyber warfare,” which is characterized by the mediatization of politics and conflicts, has become more and more prominent in the global landscape. “Cyber warfare” refers to the action of using the Internet to carry out targeted information penetration to influence the public’s beliefs, opinions, moods, and attitudes, and creating a favorable public opinion environment for the fight for political initiative and military victories (Singer and Friedman, 2014; Tabansky, 2011). Theoretical research on cyber warfare has thus far been dominated by analyses of state-centric actions, with a focus on how nation-states utilize cyber tactics to advance their national interests in areas such as national defense, diplomacy, and military operations (Maskun and Rum, 2021; Renaud et al., 2022). Scholars have predominantly examined the strategic applications of cyber warfare from the standpoint of information warfare, disinformation campaigns, and the manipulation of media narratives to achieve geopolitical objectives. These studies have greatly enriched our understanding of cyber warfare as a tool for statecraft, particularly in the context of state and non-state actors engaging in a new form of geopolitical conflict (Arquilla and Ronfeldt, 1993).

In practical terms, existing research has largely focused on the role of national governments and the media in shaping the discourse and execution of cyber warfare strategies (Lambert, 2022). Scholars have explored how cyber warfare is employed as an extension of state military and diplomatic capabilities, with research often highlighting the importance of digital infrastructure and cyber tools in contemporary conflict (Iasiello, 2015). Investigations into media representations of war, propaganda, and public diplomacy have revealed how governments use information framework to influence international audiences and shape public perception (Szostek, 2020; Taverner, 2010). Furthermore, scholars have explored the use of digital platforms in diplomatic negotiations, espionage, and cyberattacks, emphasizing the effectiveness of these tools in advancing state-level objectives (Gartzke, 2013). However, while extensive attention has been paid to these applications, there has been relatively little focus on how individual political leaders utilize cyber platforms to perform and project their leadership in wartime scenarios.

In contrast to the predominantly state-focused or media-driven analyses in literature work, our study introduces a more comprehensive, systematic analytical framework that focuses on the individual political leader’s political performance in the context of cyber warfare. The innovative aspect of this research lies on its multi-dimensional approach. It integrates content analysis, emotional rhetoric, and social network analysis. Specifically, we employ content analysis to examine the thematic framing of war narratives, while focusing on how leaders like Zelenskyy use war narratives to enhance political image during wartime. By employing case study methodology, we delve deeper into performance strategies, illustrating how leaders manipulate digital platforms to convey emotions, such as empathy and heroism. Moreover, our research extends into audience reception through sentiment analysis and social network analysis, enabling us to evaluate the emotional and relational dynamics between the leader’s performance and public reaction. This multifaceted approach allows us to offer new theoretical insights into the specific role of political leaders in shaping international public opinion during cyber warfare, bridging gaps in the current understanding of leadership’s symbolic and performative power in the digital age.

### 2.2 Social media political performance by political figures

Leaders have long recognized the importance of communicating and cultivating an image to help maintain power and order. In the media practices of leadership, political leaders often use political performance to foster connections with their followers. The concept of “political performance” can be traced back to Erving Goffman’s classic work “The Presentation of Self in Everyday Life,” where he introduced the idea of “impression management” (Goffman, 1956). This concept involves individuals presenting themselves and their behaviors to others, guiding and controlling the impressions others form of them. In dramaturgy theory, Goffman indicated that actors (leaders) shape the audience’s perception of situations through stage performances, thereby defining the context. That is to say, leaders within a cultural environment construct politics through compelling narratives to meet the demands of political communication. Gardner and Avolio (1998) interpreted the construction of leadership significance during conflict events as a form of theatrical performance. Leadership was thus emphasized as an internally constructed discourse phenomenon involving complex interactions among leaders, followers, and contextual factors (Hamilton and Bean, 2005). With the rapid expansion of social networking sites such as Facebook, Twitter, and YouTube, discursive strategies and performative tactics stemming from the media are now everyday practices in the presentation of the self and interactions in social life. The legitimization of a leader’s role is constructed on the basis of political status and the leader’s charisma. Storytelling has become a powerful tool for leaders to negotiate their roles.

In 2022, the “Digital 2022 Global Overview Report” released by We Are Social and Hootsuite showed that there are nearly 5 billion social media users worldwide, with Twitter playing an important role in news dissemination. On one hand, social media has provided new opportunities to transform political communication (Duncombe, 2019a). Political figures now have unprecedented chances to shape narratives on the international stage and engage directly with their followers to negotiate the meaning of their leadership, especially during periods of heightened political activity. Patrikarakos (2017) found that social media can mobilize public support to pressure foreign governments into taking action against combatant forces involved in regional conflicts. Some scholars observed that during times of social upheaval, leaders increase their social media activities and shift public attention from domestic policy issues to foreign policy matters, strategically diverting focus when their positions may be at risk (Barberá et al., 2024; Barberá and Zeitzoff, 2018). On the other hand, digitization is transforming diplomatic communication and practice (Bjola and Holmes, 2015). Social media bridges the gap between political figures and citizens to form communication patterns centered around interaction and engagement. Shi and Tong (2020) argued that digital public diplomacy has served as a crucial means for states to convey their voices to the international community, strengthen international exchanges, and foster cooperation. Essentially, it aims to influence and persuade the foreign public, building positive and enduring relationships with the audience (Shi and Zhang, 2020). Digital public diplomacy therefore becomes a popular strategy to target different audiences and mobilize public support for foreign policy by increasing transparency and opportunities for public engagement.

In exploring the strategies of political figures’ social media performances, Goffman categorized impression management strategies used by public figures into four types: idealization, mystification, debunking, and remediation. Building upon dramaturgy theory, Aggestam and Hedling (2020) examined how particular frames assisted the performance of Mogherini’s leadership through attention to the interaction order, impression management, and copresence. They developed a new leadership frame based on three key concepts to analyze specific leadership strategies. Additionally, in studies on new pathways of political communication in the era of smart media, Shi and Pan (2023) identified key strategies employed by political figures in Western countries on social media platforms, including constructing “chains of interaction rituals,” amplifying the effects of “metaphorical framing,” and leveraging “emotional arguments.” Previous research by scholars indicates that the performance of leadership during conflict events is closely related to the strategies adopted by political leaders. These strategies can shape leadership roles and images, mobilize public opinion, and ultimately serve political purposes effectively.

### 2.3 War narrative frame construction and communication effect

Framing theory refers to the reflection of reality, the construction of meaning, and the guidance of cognition through text and discourse (Goffman, 1974). In order to view political performance as meaningful actions established by political leaders themselves, we drew on Goffman’s perspective and used “frame” as an analytical tool. In the field of mass communication research, framing analysis has been identified as aspects of perceiving reality and making it more salient in communicating texts, improving problem definition, moral evaluation, causal interpretation, and response recommendations with selective and salient influences (Entman, 1993). Message framing is an important part of campaign strategy, and social media campaigns are no exception. Cai and Liu (2022) pointed out that media presentation of “facts” about war, support for war information activities, and the media’s approach to war all influence societal frames for understanding and responding to war. In media framing studies, Semetko and Valkenburg (2000) summarized five generic frames for reporting on European political issues: conflict frame, human interest frame, economics frame, morality frame, and responsibility frame. Building on this foundation, Lecheler and De Vreese (2012) added a factual frame and leadership frame. Many scholars believe that general frames are universal elements of mediated political messages. Frame building has incorporated various linguistic devices such as metaphors, exemplars, catchphrases, and depictions (Gamson and Modigliani, 1989; Van Gorp and van der Goot, 2012). We apply frames to transform social realities into subjective perceptions, aiding in defining leadership in political performances as compelling rhetoric. This is crucial for mobilizing audiences and achieving consensus.

With the deepening study of metaphorical relationships, many scholars have recognized the framing function in visual semiotics practices, leading to the exploration of the mechanisms of visual meaning generation. Griffin (2004) discussed the nature of American news photographs related to the “war on terrorism” during the Afghanistan and Iraq wars, exploring the issue of news photographs as a form of news framing. He argued that these photographs primarily serve to establish the narrative subject of official discourse. Manor and Crilley (2018) supposed that images play an important role in the interaction between narratives and frames. Resonance with narratives often requires frames conveyed through contextual and typically timeless dimensions, as depicted in imagery. Hu and Li (2024) pointed out that images generate meanings in novel ways, altering the scale and magnitude of information flow. In the shift toward visual culture and innovation in media forms, they highlight the loose coupling and distributed interactive relationships of media carriers in information dissemination. Integrating visual frames into the analysis of war narrative frames provides a frame to deeply explore war phenomena, enriching research perspectives and dimensions.

In previous research, exploring communication effects has aided in better analyzing the effect of communicators’ strategies. The media’s communication effect reflects to what extent communicators achieve their intended goals or objectives during their communication activities (Zhan, 2018). Price and Tewksbury (1996) observed that different frames may generate varying short-term and long-term effects when strategically constructing frames to capture the audience’s mindset. In the current quantitative research paradigm, researchers typically use easily accessible data to measure and analyze these effects, such as retweets, shares, and comments, to observe audience engagement on social media. These data are particularly intuitive for assessing short-term effects. Additionally, scholars found that the actual content of tweets and mentions of key relevant figures are critical factors influencing the effect of Twitter communication (Yang and Counts, 2010). In fact, user-generated content serves not only as a key indicator of communication effects and interaction quality but also as a powerful manifestation of the ability to shape public opinion and set agendas. This study aims to further explore the network agenda-setting effects of social media, with a view to comprehensively and meticulously revealing the communication effects of political figures using social media.

**Figure 1:**
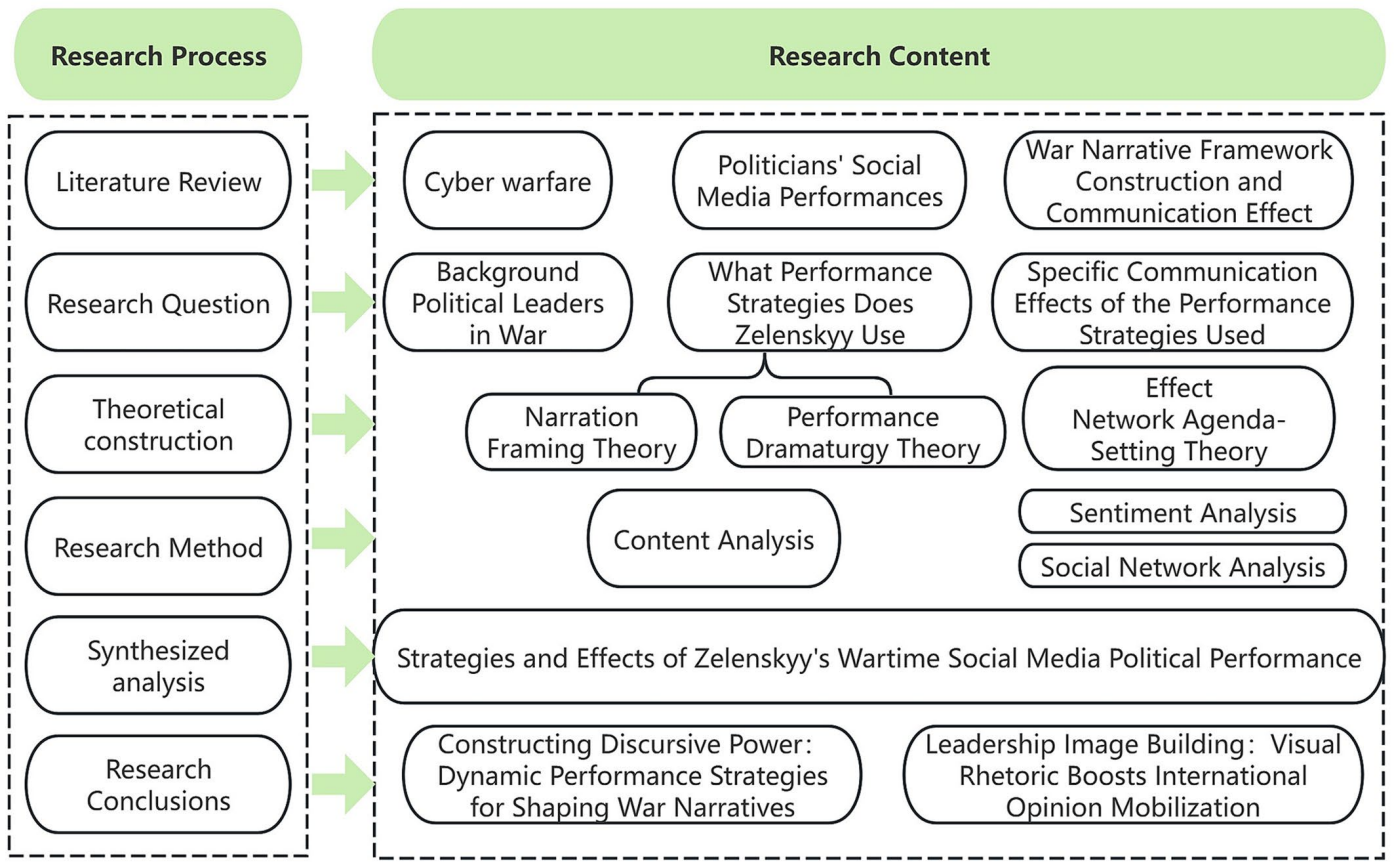
Research framework diagram.

Based on the previous context, we present the following three research questions and the overall research framework diagram (Figure 1).

*RQ 1*: How does Zelenskyy strategically use Twitter to respond to and guide public opinion on the Russia-Ukraine war? What war narrative frames does he construct?

*RQ 2*: Throughout Zelenskyy’s political performances amid the war, what characteristics does the war narrative frames he employed exhibit over time? What specific political performance strategies does he use?

*RQ 3*: How is the effect of the war narrative frames constructed by Zelenskyy?

## 3 Materials and methods

### 3.1 Data collection and preprocessing

This study focuses on Zelenskyy’s political performance strategies on social media and their effects. We primarily examine three variables: Zelenskyy’s narrative strategies, performance strategies, and audience feedback. Twitter is a platform that allows users to share text, images, videos, and links with their “followers,” characterized by immediacy, interactivity, and multimedia features. Moreover, numerous studies have shown that Twitter has been widely used by politicians as a paramount tool to political strategize communication (Manor and Crilley, 2018; Soedarsono et al., 2020). On Twitter, Zelenskyy’s narratives are presented through the text content of his tweets, while his performance strategies are enhanced through images or videos. Audience feedback is reflected in the platform’s interactive features such as likes, retweets, and comments. Therefore, compared to other news media platforms, we have chosen Twitter as the source of data for this study.

To collect data from the Twitter platform, we first established the time frame for data sampling. The Russia-Ukraine war began on February 24, 2022, after which Zelenskyy posted a large number of tweets related to the conflict. Over the following year, the war gradually entered a state of normalization, showing no signs of a quick resolution. Therefore, we ultimately chose to collect Twitter data from February 24, 2022, to February 23, 2023. In June 2023, we used Twitter’s Academic API to gather all tweets from Zelenskyy’s account (@ZelenskyyUa) within the specified time frame. This was achieved by making API calls in Python using private tokens and keys for authentication. Considering the international significance of the event and its relevance to our research objectives, we selected only the English tweets as our research sample. We then collected the first 100 comments for each of Zelenskyy’s tweets. Some tweets may have fewer than 100 comments. By using the “conversation_id,” we linked the comments to their corresponding Zelenskyy tweets to ensure data integrity and traceability.

After deduplication, 604 valid tweets and 58,100 corresponding comments were obtained from Zelenskyy’s Twitter account. The Twitter API provides access to the raw data of tweets, which includes a comprehensive range of information. We selected only the fields relevant to this study, including the time of publication, text content, images, videos, number of comments, retweets, likes. For the comments on the tweets, we retained only the “author_id” related to the user, excluding any other private data.

Similar to many studies, such as Boon-Itt and Skunkan (2020), we carried a substantial work to process and filter the tweets before conducting data analysis. We preprocessed the Twitter data, including data cleaning, stop word filtering, and lemmatization, which are common techniques in tweet analysis. First, we used regular expressions in Python to remove noise characters from the tweets, including URLs, emojis, and other non-English characters. Then, we utilized the Natural Language Toolkit (NLTK) library for lemmatization. Finally, we filtered out stop words from the tweets based on the English stop word dictionary provided by NLTK. We checked the preprocessed data and found no tweets with empty content or other anomalies. Ultimately, the preprocessed data was used in subsequent data analysis.

### 3.2 Methods

#### 3.2.1 Content analysis

Content analysis is a research method that objectively, systematically, and quantitatively describes communication content (Berelson, 1952). Based on theoretical perspectives, it converts non-quantitative content such as text (or images, videos, etc.) into quantitative data through coding, enabling quantitative analysis of research content and factual judgments. Framing analysis studies focus on the relationship between public policy issues in the news and public perceptions of those issues (Güran and Özarslan, 2022). The use of frame analysis as a reliable indicator of content analysis helps to explore the evolution over time of the specific performance strategies employed by Zelenskyy. The study is first conducted using Python for latent Delicacy Allocation (LDA) theme modeling. This process generates results for the distribution associated with each topic glossary and the probability distribution of choosing a specific topic for each tweet. Using computer-aided content analysis we extract the most characteristic topics and their 20 most frequent words from tweets. However, since it is difficult to achieve effective coding by machine alone, we use a combination of machine and manual methods to inductively determine the topic frame based on reference to common generic frameworks. Ultimately, six subcategories under two main categories (textual and visual) of frame themes are identified for tweet coding (Table 1).

**Table 1:** Representation of frame building and its operational definition.

Variable	Operational definition	Example
Leadership frame	The message emphasizes personal leadership actions as a political leader	I want to wish all of us one thing – victory. And that’s the main thing. Glory to Ukraine! Difficult, but so important and necessary VICTORY!
Diplomacy and international support frame	The messages document the diplomatic mediation efforts of various countries, expressing gratitude for specific assistance provided to Ukraine	Sweden provides military, technical, and humanitarian assistance to Ukraine Belgium is sending us another 3,000 machine guns and 200 anti-tank grenade launchers
Conflict and opposition frame	The message mentions conflict between countries and/or emphasizes disagreement among, between, or with them The message attacking or reproaching Russia uses inflammatory and emotional language to condemn Russia’s war crimes	Russian atrocities in the Kyiv region must be investigated and Russia itself must face new painful sanctions RF terrorists remain terrorists. #RussiaIsATerroristState
Image construction frame	Pictures or videos visually portray a positive and strong leader	Personal speeches, participation in international conferences, and related images or videos of diplomatic activities
Morality and responsibility frame	Pictures or videos depict post-war scenes	Casualties and city ruins in Ukraine
Endorsement frame	Pictures or videos endorse the textual content	The text emphasizes Ukraine’s contribution to food security, accompanied by an image of grain transport ships

Before the formal frame coding, two Master’s students majoring in journalism and communication undergo reliability testing during the pre-coding phase. Upon examination, Cohen’s kappa values for the categories of war narrative textual and visual frames are 85.8 and 86.3%, respectively, meeting the standards for content analysis.

#### 3.2.2 Sentiment analysis

Sentiment analysis refers to the process of analyzing, processing, and extracting subjective texts with emotional content using natural language processing and text mining techniques. We use Flair, a tool based on deep learning algorithms, for sentiment analysis, rather than rule-based techniques. Flair^1^ use pre-trained contextual embeddings to capture the semantic meaning of words and phrases in context (Kaur et al., 2024). To better explore the effect of Zelenskyy tweets on public sentiment, we use Flair to score the sentiment of the top 100 public comments under Zelenskyy tweets. Then, we identify positive comments with a threshold of 0.8 certainty, and quantifying the positive sentiment scores of Zelenskyy tweets by calculating the percentage of positive comments.

#### 3.2.3 Social network analysis

Social network analysis, as a method for studying social relationships, is commonly used to describe and measure relationships between actors or to map and measure relationships and flows between different nodes (Hanneman and Riddle, 2005). Previous scholars have applied social network analysis to the research field of agenda-setting to provide a more detailed and enriched understanding of media and public agendas by sorting out the network relationships between the various elements emphasized in news reports and other communication channels (Guo, 2012; Guo and McCombs, 2011). In order to more effectively explore the effect of Zelenskyy’s political performance strategy, we include network agenda setting effect analysis in our study. Social network analysis is used to identify issue network relationships between tweets posted by Zelenskyy’s account and tweets commented on by the public. The specific steps are divided into three categories according to the text frames, calculate the co-occurrence of media tweets and public tweets, respectively, in terms of a single tweet, use the Quadratic Assignment Procedure (QAP) toolkit^2^ in Python, take the media agenda matrix as the X variable and the public agenda matrix as the Y variable, and set the number of iterations as 2000 to calculate the degree of correlation between media agenda and public agenda. After assigning values to the number of issue co-occurrences the media agenda network and public agenda network are presented visually using Gephi.^3^

## 4 Results and findings

We first focus on Ukrainian President Volodymyr Zelenskyy’s use of social media during wartime. The construction of the frame represents the leader’s political stance and perspectives on issues. We conduct a descriptive analysis of the sample tweets, aiming to reveal how Zelenskyy strategically uses Twitter to address and influence war-related public opinion, addressing RQ 1. Subsequently, based on the influence characteristics of retweets, likes, and comment data, we categorize Zelenskyy’s use of social media into stages and then summarize his war performance strategies through case studies to answer RQ 2. Finally, this study evaluates the phased performance of Zelenskyy’s constructed war narrative frames in setting the agenda on social media, providing empirical evidence for the effect of social media in guiding public opinion, thereby addressing RQ 3.

### 4.1 Frame construction: Zelenskyy’s use of social media during wartime

**Figure 2:**
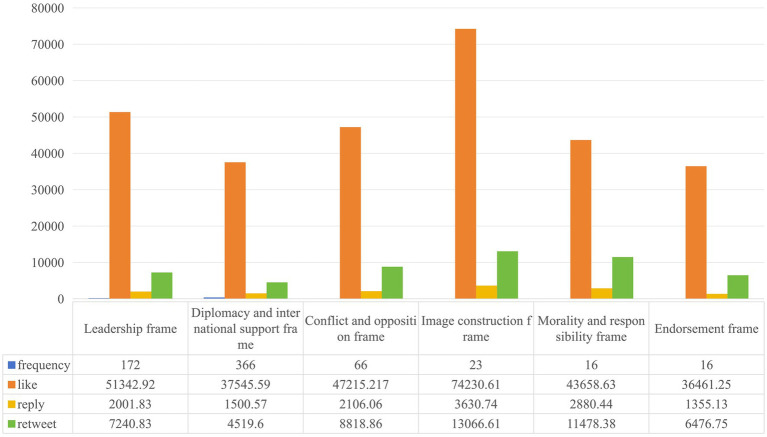
Statistical results of Zelenskyy’s constructed war narrative frame.

Based on Figure 2, it is evident that Zelenskyy primarily constructs war narrative frames using text in his tweets. The most frequently used frame is diplomacy and international support (60.6%), followed by leadership (28.5%), with the conflict and opposition frame being relatively less used (10.9%). Visual frames are used less frequently, primarily focusing on image construction (41.8%). In terms of engagement, the public tends to prefer liking and commenting on tweets that showcase Zelenskyy’s positive leadership image.

We conduct positive sentiment scoring for each public comment on every tweet. The data satisfies the assumptions of normal distribution and homogeneity of variance and we use ANOVA. The results show a significant difference among positive sentiment scores under different war narrative frames (*p* = 0.046 < 0.05). *Post-hoc* LSD analysis indicates that tweets framed around leadership and diplomacy and international support elicit significantly higher positive emotions from the public compared to tweets framed around conflict and opposition. This suggests that comments under tweets framed in a conflict-oriented manner tend to be more negative overall.

**Figure 3:**
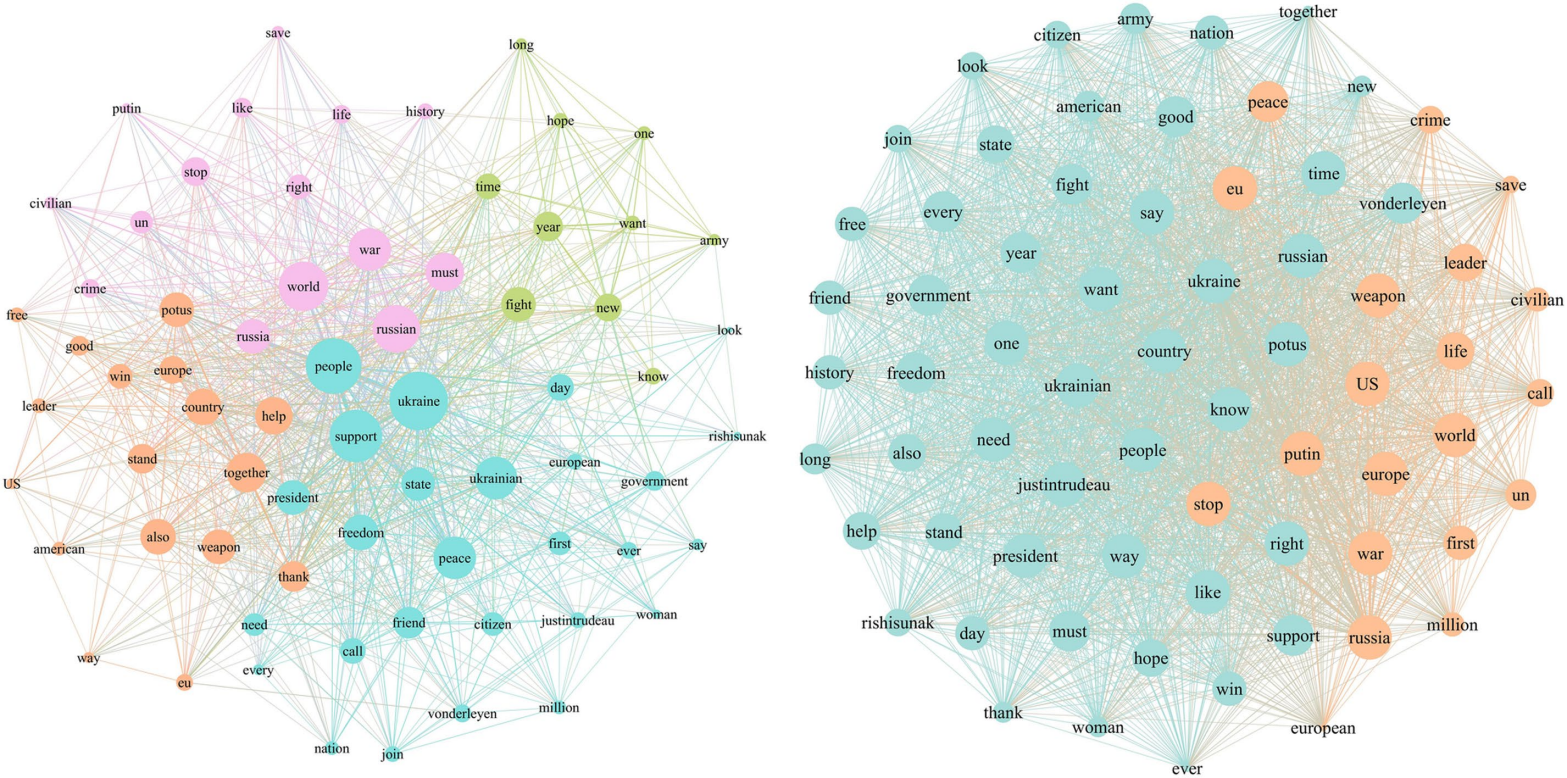
Media agenda and public agenda of leadership frame.

The agenda networks of text-based war narrative frames are visualized to illustrate relationships between attributes. In the visualization, each node represents an attribute, with nodes positioned more centrally indicating greater connections with other attributes. Larger nodes indicate a higher degree centrality, suggesting they are more central to the discussion topics. Based on Figure 3, in the use of the leadership frame, topics such as “Ukraine,” “people,” “support,” and “world” are at the core of the media agenda and are frequently mentioned. As the President of Ukraine, Zelenskyy actively constructs topics like “nature of the war,” “sanctions against Russia,” and “hope for victory” to influence public opinion. In the public agenda, the topics are more dispersed, with frequent mentions of political figures involved in the conflict and stakeholders. Additionally, positive topics such as “like,” “help,” “rescue,” and “support” are quite prominent.

**Figure 4:**
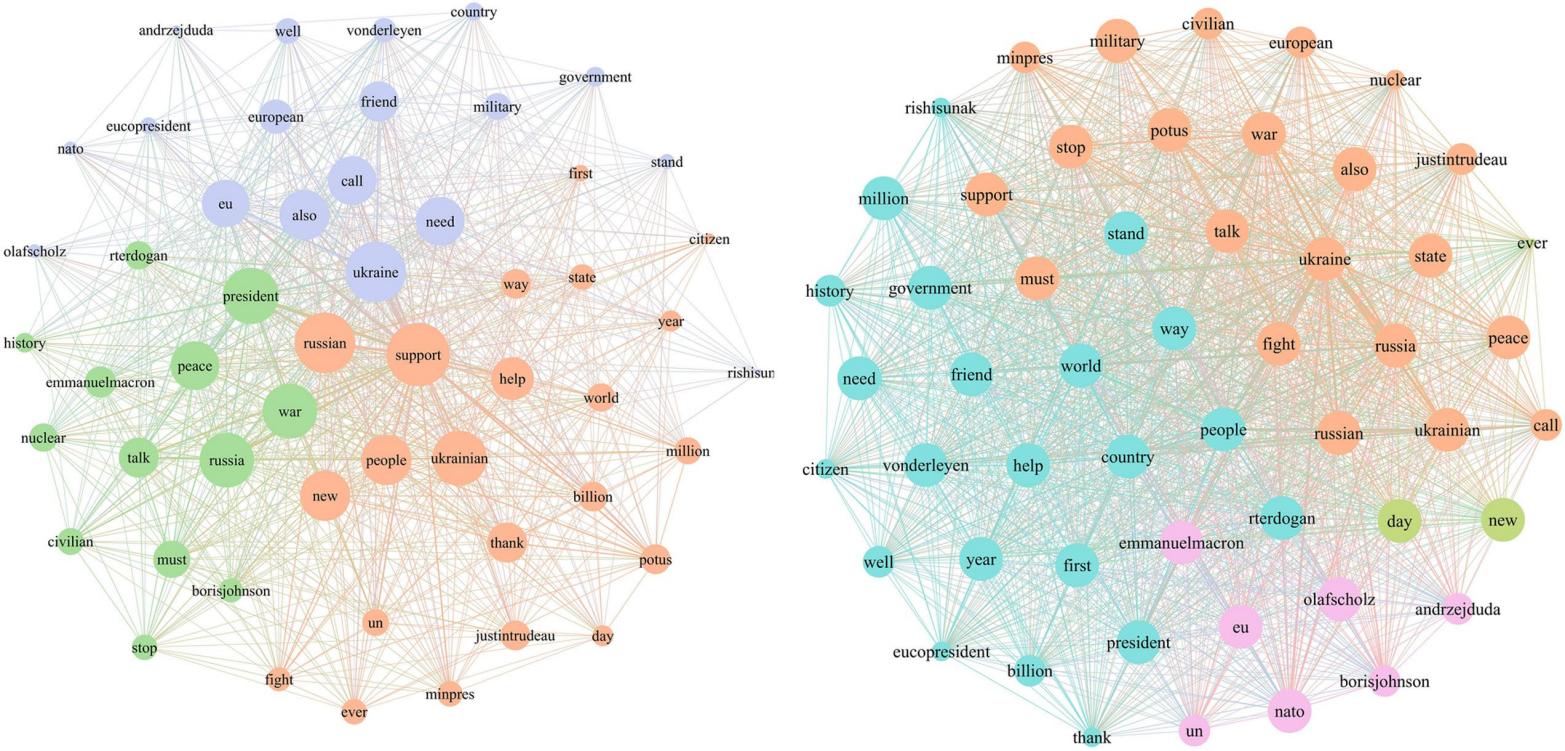
Media agenda and public agenda of diplomacy and international support frame.

From Figure 4, we can see that in the diplomacy and international support frame, the attribute “support” clearly occupies the central position in the media agenda. Topics such as “talk,” “president,” “call,” and “thanks” are concentrated, indicating Zelenskyy’s proactive efforts to secure international allies and pressure on Russia. In the public agenda network, there is a high similarity in comments, with significant attention to topics like “ceasefire” and “peace.”

**Figure 5:**
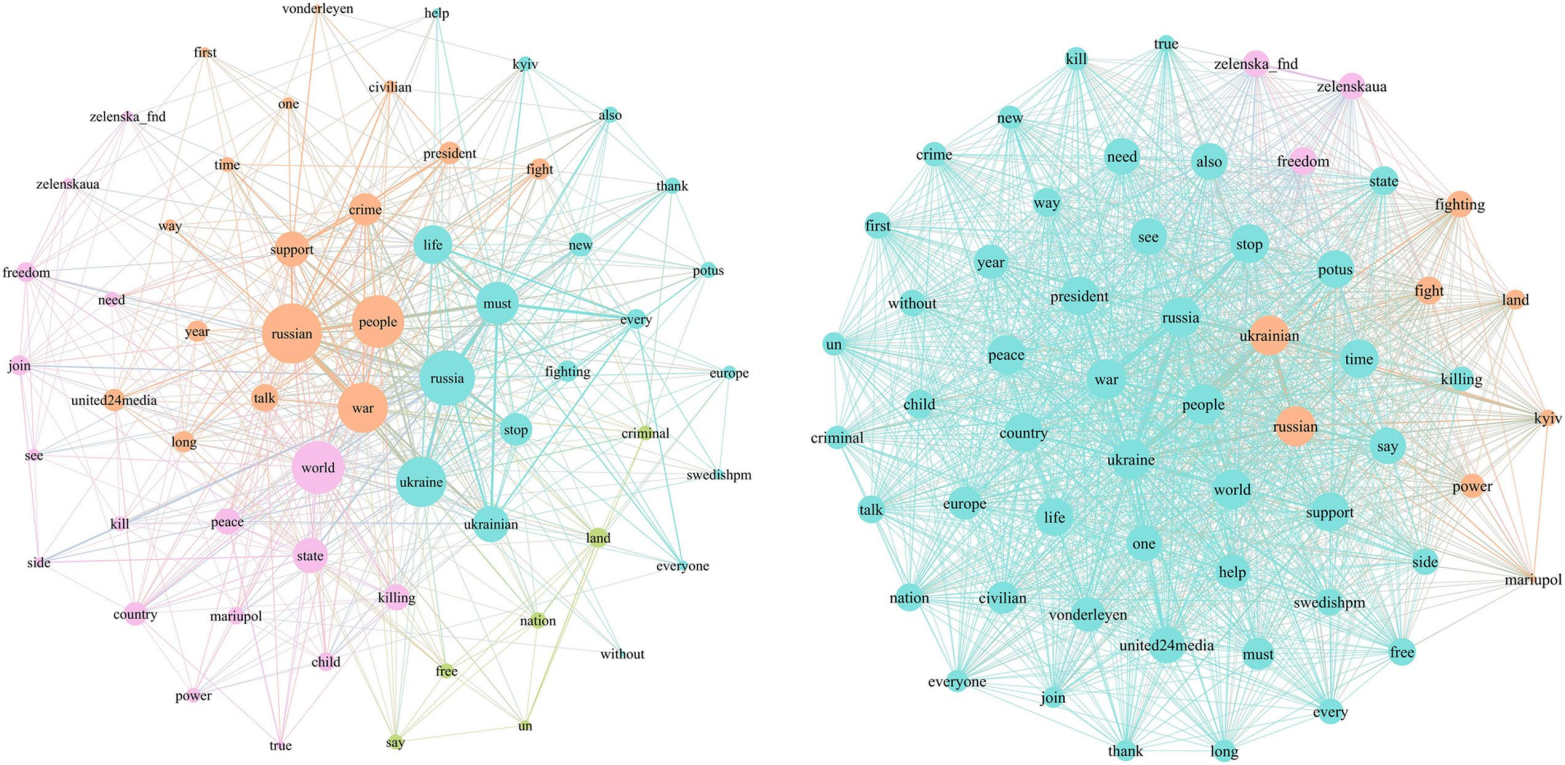
Media agenda and public agenda of conflict and opposition frame.

Based on Figure 5, in the conflict and opposition frame, Zelenskyy connects “Ukraine” with attributes such as “fight,” “stop,” and “life,” emphasizing Ukraine’s fight for justice. He associates “Russia” with attributes like “battle,” “crimes,” “killing,” and “combat,” condemning Russia’s war crimes and constructing the narrative of “resisting Russia.” In the public agenda network, attributes such as “defend Kyiv” and “freedom” are more prominent.

### 4.2 Temporal evolution: sequential changes in Zelenskyy’s performance strategy

**Figure 6:**
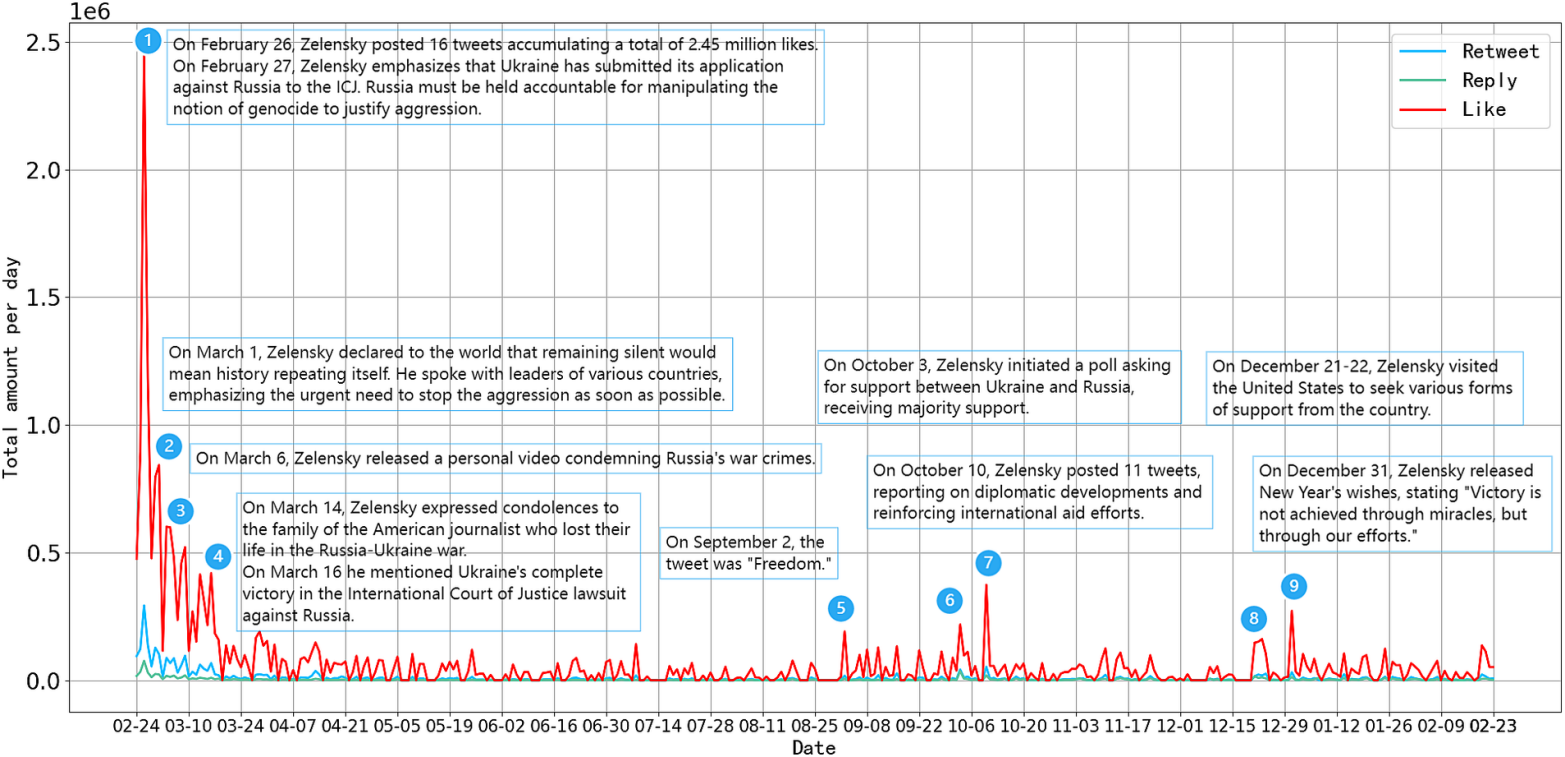
The dissemination impact of Zelenskyy’s wartime tweets.

To investigate the effect of tweet dissemination, we aggregate the retweets, likes, and comments data of daily tweets in this study sample (Figure 6). Volodymyr Zelenskyy’s tweet engagement experience a burst of growth early in the war, reaching its peak on February 26, 2022. The Russia-Ukraine war is the first large-scale geopolitical conflict of the social media era, attracting significant international attention to its progression. This period also marks the peak of Zelenskyy’s tweet postings, where the actions of political leaders have a significant impact on shaping international perceptions and attitudes toward the war. As the conflict continued, the overall dissemination of tweets slows between April and August, with minor fluctuations, indicating a general decline in international public attention during this period. Two minor peaks in dissemination occur from September to November and December to February. The first peak corresponds to the Ukrainian counteroffensive on the battlefield, where changes in the war’s dynamics may spur a temporary increase in dissemination. The second peak coincides with Zelenskyy’s visit to the United States and the New Year, both of which rekindle widespread interest in his tweets. From the perspective of dissemination effectiveness, we divide Zelenskyy’s social media performance during the first year of the conflict into four stages.

#### 4.2.1 Stage 1: visual metaphor—construction of heroic imagery in wartime

Lakoff and Johnson (2008), in their book “Metaphors We Live By,” have pointed out that “metaphor is a systematic mapping across conceptual domains.” Metaphors fundamentally guide the understanding of one concept in terms of another, leading to the subjective construction of the target concept. Zelenskyy leverages this aspect of social media imagery to construct visual metaphors of himself during the war. On March 6, 2022, Zelenskyy posted a video tweet with the caption “Breaking.” In the video, he crafted an “imperfect” and “vulnerable” image of president by appearing in a signature military green T-shirt and sporting a full beard.^4^ In the seven visual tweets from the early stages of the war, this image recurs frequently. The green T-shirt metaphorically represents the Ukrainian army, militia, and civilians, making it easy for the public to associate him with the armed forces amidst the conflict. This visual symbol non-verbally conveys that Zelenskyy is closely monitoring the frontline situation. His unkempt appearance resonates with the instability and anxiety experienced by the populace during wartime. Through visual metaphors and the cognitive narrative of being present in the war, Zelenskyy transitions his image from a political elite to a “people’s guardian” and “heroic president.”

Sentiment analysis of public comments reveals a significant difference in public emotions across different war narrative frames during Stage 1 (*p* = 0.00 < 0.05). According to LSD *post-hoc* analysis, the positive sentiment scores in the comments under the leadership frame and the diplomacy and international support frame are significantly higher than those under the conflict and opposition frame. This suggests that during Stage 1, Zelenskyy’s statements as a political leader are more likely to garner the international audience’s attention and positive emotional support.

**Figure 7:**
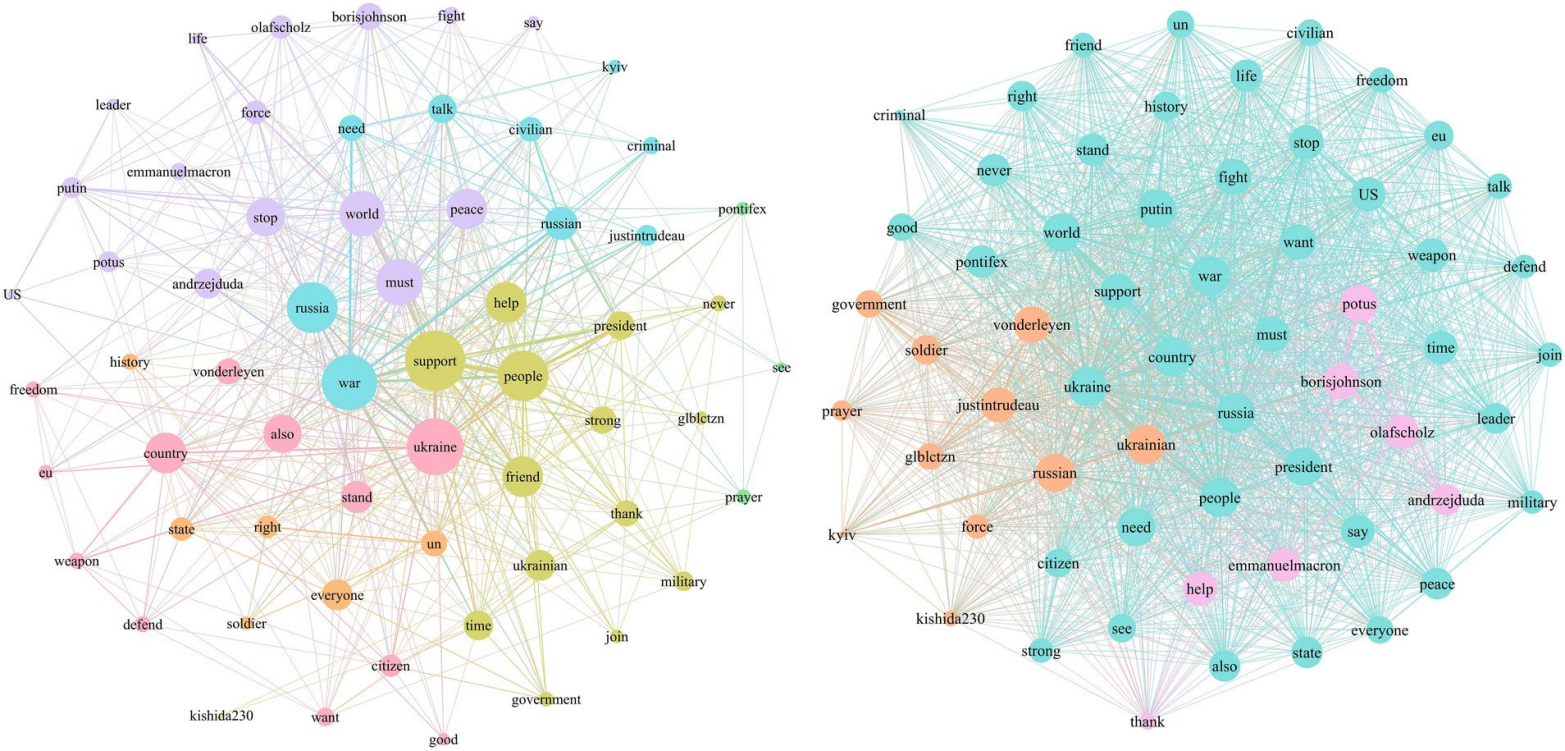
Stage 1: media agenda and public agenda of leadership frame.

In the early stage of the war, Zelenskyy catered to the wartime needs of Ukraine and Western politics by portraying himself as a “national hero” and a “tragic figure.” As shown in Figure 7, in the agenda attribute network for the leadership frame during Stage 1, topics such as “support for Ukraine” prominently occupy the core of the media agenda. These topics are linked with attributes such as “help,” “friend,” “president” and “thank,” constructing narratives around “international assistance” and “stop Russia.” The combination of verbal and non-verbal communication made Zelenskyy ubiquitous in both Ukrainian and Western public spheres. This not only earns him the trust of many countries’ citizens but also makes Ukraine unprecedentedly united, continually reinforcing his “hero” image.

#### 4.2.2 Stage 2: media diplomacy—performance and spectatorship on social media

According to Gainous and Wagner (2014), political actors can autonomously produce and directly share information with the public through social media, influencing the public agenda and achieving significant social effects by creating and framing narratives. Social media constructs a space for dialog, enabling politicians to engage in effective impression management and garner public support. Meanwhile, the public participates in this new arena of political communication through watching and interacting. The interplay between political messaging and public interaction exemplifies how social media serves as a powerful tool for shaping political discourse, highlighting the importance of narrative framing in achieving desired social effects.

**Figure 8:**
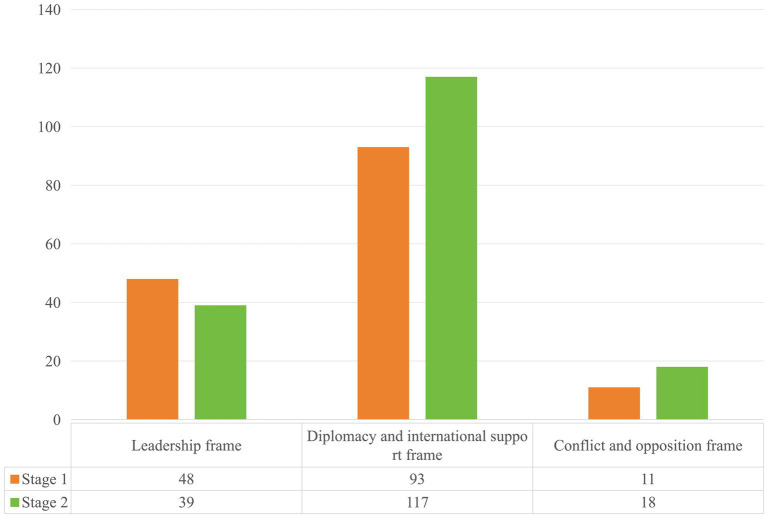
Differences in the use of war narrative frame between stage 1 and stage 2.

**Figure 9:**
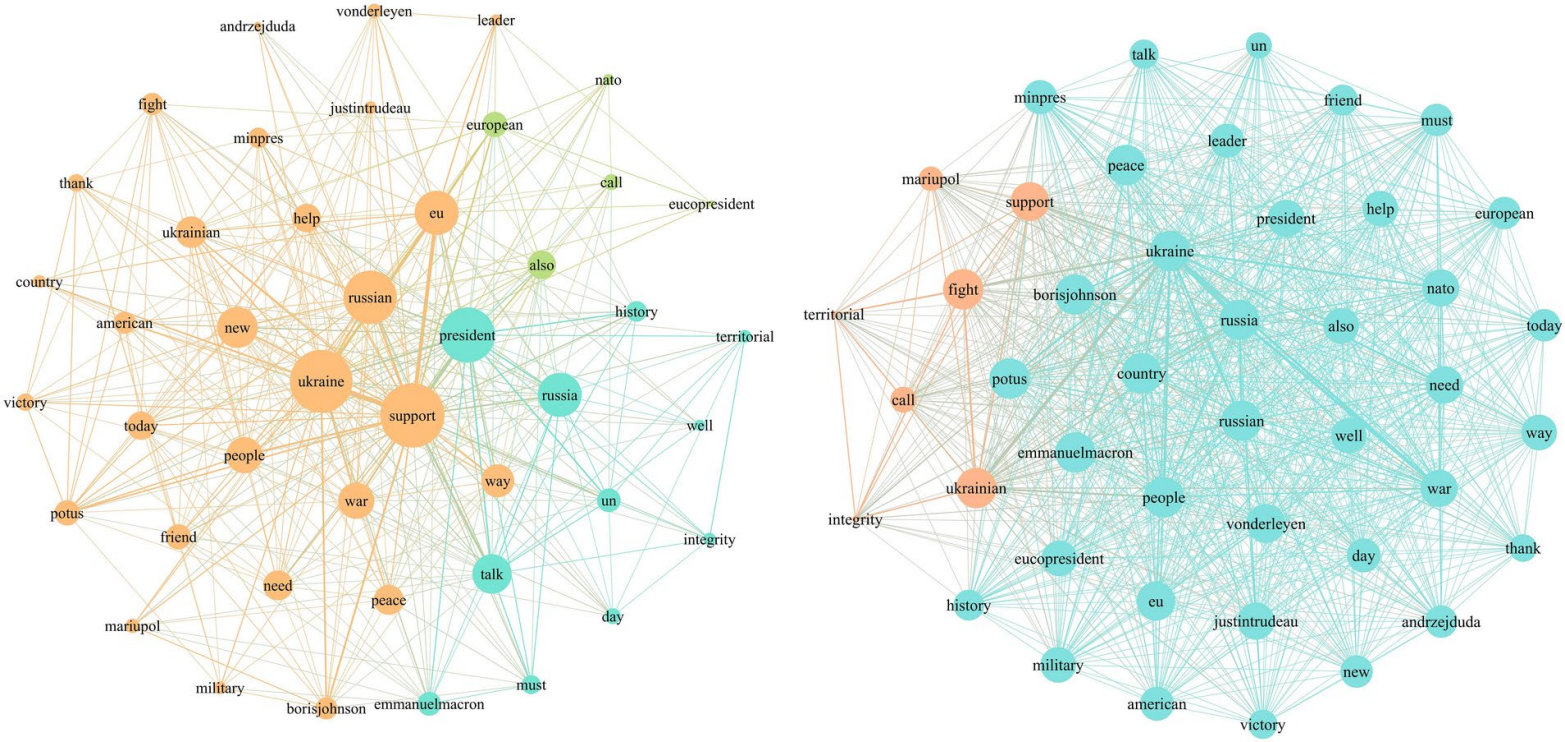
Stage 2: media agenda and public agenda of diplomacy and international support frame.

Figure 8 shows that in Stage 2, Zelenskyy’s tweets continue to predominantly use the diplomacy and international support frame, with enhancements compared to Stage 1. In Figure 9, Zelenskyy’s narrative themes within the diplomacy and international support frame become more focused, with “supporting Ukraine” positioned as a central topic. Starting in April, Ukraine shifted from comprehensive defense to focused defense. During this stage, Zelenskyy focuses on garnering support from the West, emphasizing media diplomacy. He actively tweets real-time updates on war mobilization, battlefield situations, speeches, records, and international mediation efforts. These efforts not only capture global public attention but also garner morally coercive international support. Furthermore, Zelenskyy continuously tweets thanks to leaders and governments worldwide, reinforcing a unified Western image in response to Russian aggression and significantly amplifying the impact of these aids.

#### 4.2.3 Stage 3: emotional argument—humanizing to evoke international empathy

Classic rhetorical theory of effective persuasion includes three forms of argumentation: logical argumentation, relying on evidence and reasoning; ethical argumentation, based on the speaker’s character, isdom, moral qualities, and goodwill; and emotional argumentation, utilizing emotional appeals to enhance persuasive effect (Griffin, 2006). The visual and fragmented expression on social media platforms amplifies the advantage of emotional argumentation, providing a more rapid and effective means of invoking response and fostering empathy. This shift toward emotional argumentation is particularly relevant in the digital age, where political figures leverage visual imagery to evoke strong emotional connections with their audience.

Ukrainian President Zelenskyy has brought the skills of an actor into the current wartime politics. Deep within the vortex of war, Zelenskyy cleverly employs the humanization and empathy strategy in his tweets, combining international historical events to evoke public emotional resonance. On September 8, 2022, Zelenskyy tweeted sincere condolences on the passing of Queen Elizabeth II of the United Kingdom;^5^ on the 11th, he paid tribute to the victims of 9/11 in the United States, emphasizing that terrorism has no place to thrive.^6^ In addition, during this stage, he primarily uses the victim narrative frame visually, highlighting the tragic post-war situation in Ukraine. On September 26, 2022, Zelenskyy tweeted “Kharkov. Saltivka A large, quiet neighborhood. It was like this until the Russian invasion.” in stark contrast to the accompanying photo, the devastated ruins of the city.^7^ He elevates the defense of Ukraine to the defense of universal human values, exposing the brutality of war while reinforcing the narrative of victory in war. By invoking emotions of fear, confidence, outrage, and compassion, he further strengthens the persuasive effect.

#### 4.2.4 Stage 4: interaction ritual chain—the interactive ritual of short videos

American sociologist Collins (2004) introduced the theory of Interaction Ritual Chains in his book “Interaction Ritual Chains.” Drawing from this theoretical model, it has been found that on platforms like Twitter, short videos create ritual interactions where global audiences converge around shared focal points provided by the videos. The comment sections serve as spaces for collective interaction. This construction of “Interaction Ritual Chains” through the dissemination of short videos fosters emotional resonance among the public, stimulates a sense of belonging and identification, and thereby replenishes emotional energy. This strengthens the effect of “zero-distance” communication under the interactive order. By drawing on Collins’ Interaction Ritual Chains, we can better understand how Zelenskyy’s strategic use of social media deepens the emotional engagement of his followers and reinforces a shared sense of purpose in the context of the ongoing conflict.

In two tweets posted by Zelenskyy on January 11 and January 20, 2023, unlike traditional political leaders who appear in formal attire, Zelenskyy opted for a casual black long-sleeved sweater, presenting a simple wartime image.^8^ In his speech videos, Zelenskyy often started with phrases like “Dear Ukrainians, I wish you health!” or “Today, as always, Ukraine honors,” using a warm tone to closely associate himself with the Ukrainian people, thus enhancing the experience of emotional sharing. It can be observed that through a series of verbal and non-verbal symbols, Zelenskyy has fostered weak ties centered around the ritual core of “defending Ukraine” among global audiences. This has successfully achieved a connection between image construction, value transmission, and emotional arousal, thereby achieving effective communication outcomes.

### 4.3 Communication effect: stage-based network agenda setting of war narrative frame

There is a significant correlation between the war narrative frames constructed by Zelenskyy on Twitter and the media and public agendas (Table 2). The agenda-setting effects of these frames exhibit different characteristics across stages of the war and changes in public opinion environments. In Stage 1, the leadership frame demonstrates prominent effects, indicating that in the early crisis, the decisiveness and actions of leaders are crucial for shaping public perception and rallying domestic and international support. In Stage 2, the continued influence of the leadership frame and enhanced effects of the diplomacy and international support frame are evident. During this stage, Zelenskyy not only reinforces his leadership image but also intensifies efforts to garner support and cooperation on the international stage. In Stage 3, the overall agenda-setting effect of the war narrative frames weakens, with the conflict and oppositional frame showing more pronounced effects, possibly related to specific developments in the war and the intensification of conflicts. In Stage 4 the agenda-setting effects of all frames on topic discussions notably decline. As the conflict between Russia and Ukraine continues, international public attention toward Zelenskyy himself and the situation diminishes. Additionally, shifts in international public opinion environments and emerging issues may also divert public attention.

**Table 2:** Network agenda-setting effects of war narrative frame.

Stage	War narrative frame	Obs. value	Significance
Stage 1: February–March	Leadership frame	0.39	0.00
	Diplomacy and international support frame	0.30	0.00
	Conflict and opposition frame	0.29	0.00
Stage 2: April–August	Leadership frame	0.38	0.00
	Diplomacy and international support frame	0.32	0.00
	Conflict and opposition frame	0.19	0.00
Stage 3: September–November	Leadership frame	0.23	0.00
	Diplomacy and international support frame	0.22	0.00
	Conflict and opposition frame	0.27	0.00
Stage 4: December–February	Leadership frame	0.17	0.00
	Diplomacy and international support frame	0.18	0.00
	Conflict and opposition frame	0.18	0.00

Overall, Zelenskyy’s war narrative frames also exhibit characteristics of staged evolution in their network agenda-setting effects. Zelenskyy’s profound understanding and adept use of Twitter as an effective political communication tool allow him to flexibly adjust his social media strategies based on changes in the Russia-Ukraine situation and public opinion. This adaptive strategy aims to garner maximum public support and international attention. These adjustments not only help bolster Zelenskyy’s personal leadership and mobilization capabilities but also guide the direction of war discourse and shape public perceptions.

## 5 Conclusion and discussion

This study systematically analyzes the strategies and communication effects of political leaders on social media in the context of wartime crises from the perspective of political communication on social media. By integrating framing theory, dramatic theory, and network agenda-setting theory, the study proposes an innovative theoretical framework that encompasses three dimensions: narrative, presentation, and effect. This framework not only fills a gap in academic research but also provides a new theoretical perspective for understanding modern political communication, particularly regarding how political leaders project leadership, manage public perception, and influence international opinion in crisis situations.

### 5.1 Constructing discursive power: dynamic performance strategies for shaping war narratives

Currently, actively setting agendas to influence the direction of international discourse is increasingly becoming a crucial source and manifestation of a nation’s political power in response to globalization needs. In the complex arena of international public opinion, social media disrupts the monopoly of traditional political propaganda over political sources, discourse, and viewpoints. This shift has tilted the focus of political communication toward narrative logic, discourse selection, the pursuit of values, and contestation of meaning, leaning toward individual societal members (Jin and Ning, 2023). Political leaders, governments, and states operate within this social media environment, wherein they continually address crises and institute damage control through platforms such as Twitter (Duncombe, 2019b). This also imposes higher demands on the social media use strategies of political leaders. In the context of the Russia-Ukraine information war, Ukrainian President Zelenskyy integrates acting skills into political performances, swiftly mastering the art of political communication during the crisis of war. His personal charisma, acting skills, and experience in television have proved to be extremely useful in politics and war (Dyczok and Chung, 2022). This also sets Zelenskyy apart from other political leaders in terms of the style and content of social media performance. The actor-turned-president Zelenskyy, has emphasized his role as a “internet actor” who is adept at using a wealth of acting techniques to establish an emotional connection with the public. Construct a narrative of national community, and enhance his political appeal (Hordecki and Nosova, 2023). In contrast, former U.S. President Donald Trump, also a performative leader, was more of a “internet maniac” person. He uses an informal, direct and provocative communication style to construct and reinforce the notion of a homogenous people and a homeland threatened by a dangerous other, with a distinctly personal note (Kreis, 2017; Tasente, 2020). In addition, while many political leaders use social media for domestic issues or campaigning (Jensen and Anstead, 2014; Park et al., 2016), Zelenskyy has expanded its use to global diplomacy, becoming a pioneer in “Twitter diplomacy.” His strategy differs from traditional diplomacy by creating a direct and emotionally charged connection with international audiences and world leaders.

The framing of media narratives profoundly influences public understanding of issues and their solutions, as well as affecting audience attitudes, perspectives, and behaviors (De Vreese, 2005). Faced with the vast territory and strong military power of Russia, Zelenskyy has opted for a more flexible approach to propaganda, shaping international discourse through the construction of a war narrative framework. He has shifted the international community’s perception of the conflict, creating a public opinion environment favorable to Ukraine (Kalinchuk, 2023). More precisely, in terms of narrative, he has constructed three interrelated thematic frameworks: leadership, conflict/hostility, and international diplomacy and support. These themes are further reinforced by the use of hashtags, which tightly link events to these frames, thereby influencing how the international community attributes responsibility in the Russia-Ukraine war. Additionally, he has personified humanitarian aid for Ukraine’s cause as a model of social and moral expectation, a phenomenon that resembles the “bystander effect” as previously discussed by scholars (Darley and Latané, 1968). The findings show that Zelenskyy strategically adjusts his communication strategies according to his political intentions at different stages of the war, effectively mobilizing the war discourse and achieving international discourse construction. In the early stages, leveraging the “primacy effect,” political figures initiate cognitive narrative strategies that have strong initial and offensive effects (Sullivan, 2019). The leaders’ own political charisma becomes a powerful weapon to win over international public opinion, and international agenda-setting is most effective at this stage. However, with the continuation of the conflict, the multiplicity of factors makes the primacy effect diminishing (Hendrick et al., 1973). In the face of declining public attention, political leaders need to shift their social media performance strategies, where social media pressure can be effective in advancing political goals such as negotiation and aid. In a time of political discourse marketization, foreign aggression, and national insecurity are powerful messages to attract attention and influence public perceptions (Steblyna, 2020). In the middle and late stages of a conflict, discourses of hope involving descriptions of the conflict or shaping victory become a narrative strategy more capable of mobilizing public political participation. Overall, the agenda-setting effects of war narrative frames gradually diminish over time. This is possibly influenced by factors such as information saturation and public fatigue in online communication (Bright et al., 2015; Liu and He, 2021).

### 5.2 Leadership image building: visual rhetoric boosts international opinion mobilization

Nowadays, social media platforms have become intersections of national interests and sources of various international conflicts. Based on the characteristics of social media platforms that place images on top of text, it is necessary to emphasize the important role of visual image construction of political leaders in war situations (Dhanesh and Rahman, 2021). In the process of exploring the symbolic power of the leader’s image, French sociologist P. Bourdieu introduced the concept of political capital (Swartz, 2003). Fesun et al. (2019) argue that the level of popularity and trust is the fundamental feature of the politician’s image, which shapes his political capital. In the unique context of war, international audiences are often abstract entities. The more personalized and less mysterious leaders appear the more they can evoke empathy and cheers from people. This requires leaders to actively expose the foregrounded “middle ground” to shape a down-to-earth image (Yu, 2018). In a war conflict, Zelenskyy has done this well. He effectively utilizes the global platform of public opinion shaped by the West to suppress Russia and uses his personal Twitter account as a window for image self-presentation. By packaging his wartime image and combining it with emotionally compelling rhetoric, he subverts traditional perceptions of political leadership, swiftly shaping the image of a war hero president and defender of the people in the minds of global audiences. Furthermore, previous research has shown that images ‘provide access to a range of visual experiences (Perreault and Paul, 2018), and that visual presentations, such as through photographs, can reflect and enhance the personal emotions and attitudes of the publisher, and are more likely to resonate and interact with the public (Newton, 1998). The media performance behavior of political leaders profoundly affects the international public’s understanding of state action and the shaping of the state’s image. On this cyber warfare, Zelenskyy’s constant online and media presence further aided in the creation of a personal global identity and potentially forms a collective memory for Ukraine and the West in relation to the Russia-Ukraine War (Dodonov, 2022). In today’s hyperconnected world, political leadership is increasingly defined by the ability to manage and project a compelling visual narrative, particularly in times of crisis. By mastering this digital “performance,” leaders can mobilize international opinion, generate empathy, and reinforce their legitimacy. Of course, the image-building of leaders during wartime crises not only depends on carefully crafted visual narratives but also hinges on their ability to understand audience psychology and effectively leverage the characteristics of media for communication (Son et al., 2019). Ultimately, the effect of leaders’ image-building during wartime crises is a dynamic interplay between strategic narrative construction and a profound comprehension of audience dynamics, underscoring the necessity for adaptive communication strategies in an increasingly complex media landscape.

This study proposes an innovative theoretical framework for the field of political communication on social media and offers specific operational suggestions for communication practices during wartime crises. With the advancement of technology and changes in the global political environment, the role of social media in political communication will become increasingly important. Future research should deepen the exploration of this field, building upon this study to further expand it by examining cross-platform and cross-cultural comparative studies, or by focusing on how political leaders use social media during wartime crises under different political systems. This would explore the similarities and differences in their strategies and effects, providing more universal theoretical references for the political performances of leaders on social media.

This study does have several areas that could be further improved in future research. Firstly, there are certain limitations to using comments on Zelenskyy’s tweets to construct an international public agenda. Twitter data, while valuable for understanding public opinion, does not represent the entire global population, and Twitter users alone may not be fully representative of international public opinion. Secondly, frame coding based on manual analysis may introduce subjective biases. Automated or mixed-method approaches could potentially mitigate these biases and enhance the objectivity of the analysis. Thirdly, in quantifying sentiment and communication effects, we focus solely on textual comments, neglecting the significant presence of images and emoji in the comment sections. This oversight could restrict a comprehensive understanding of international public sentiments and perspectives.

## Data Availability

The raw data supporting the conclusions of this article will be made available by the authors, without undue reservation.

## Footnotes

^1^ https://github.com/flairNLP/flair

^2^ https://github.com/lisette-espin/mrqap-python

^3^ https://gephi.org/

^4^ https://twitter.com/ZelenskyyUa/status/1500472014452273157

^5^ https://twitter.com/ZelenskyyUa/status/1567935139447205888

^6^ https://twitter.com/ZelenskyyUa/status/1568917883836661762

^7^ https://twitter.com/ZelenskyyUa/status/1574340890998591491

^8^https://twitter.com/ZelenskyyUa/status/1613126818823012352; https://twitter.com/ZelenskyyUa/status/1616406133823897602

## References

[ref1] Aggestam L. Hedling E. (2020). Leaderisation in foreign policy: performing the role of EU high representative. Eur. Secur. 29, 301–319. doi: 10.1080/09662839.2020.1798411

[ref2] Arquilla J. Ronfeldt D. (1993). Cyberwar is coming! Comp. Strateg. 12, 141–165. doi: 10.1080/01495939308402915

[ref3] Barberá P. Gohdes A. R. Iakhnis E. Zeitzoff T. (2024). Distract and divert: how world leaders use social media during contentious politics. Int. J. Press/Politics 29, 47–73. doi: 10.1177/19401612221102030

[ref4] Barberá P. Zeitzoff T. (2018). The new public address system: why do world leaders adopt social media? Int. Stud. Q. 62, 121–130. doi: 10.1093/isq/sqx047

[ref5] Berelson B. (1952) Content analysis in communication research. Free Press.

[ref6] Bjola C. Holmes M. (2015). Digital diplomacy. New York: Taylor & Francis.

[ref7] Boon-Itt S. Skunkan Y. (2020). Public perception of the COVID-19 pandemic on twitter: sentiment analysis and topic modeling study. JMIR Public Health Surveill. 6:e21978. doi: 10.2196/21978, PMID: 33108310 PMC7661106

[ref8] Bright L. F. Kleiser S. B. Grau S. L. (2015). Too much Facebook? An exploratory examination of social media fatigue. Comput. Hum. Behav. 44, 148–155. doi: 10.1016/j.chb.2014.11.048

[ref9] Bronstein J. Aharony N. Bar-Ilan J. (2018). Politicians’ use of Facebook during elections. Aslib J. Inf. Manag. 70, 551–572. doi: 10.1108/AJIM-03-2018-0067

[ref10] Cai R. Liu Y. (2022). From the “twitter revolution” to “WarTok” — how social media is reshaping modern warfare. Explor. Free Views. 38, 68–78+178. doi: 10.3969/j.issn.1004-2229.2022.11.014

[ref11] Ciuriak D. (2022) The role of social media in Russia’s war on Ukraine. Available at: https://ssrn.com/abstract=4078863.

[ref12] Collins R. (2004). Interaction ritual chains. Princeton: Princeton University Press.

[ref13] Criado J. I. Martínez-Fuentes G. Silván A. (2012) Social media for political campaigning. The use of twitter by Spanish mayors in 2011 local elections. In: Web 2.0 technologies and democratic governance: Political, policy and management implications. eds. Reddick C. G. Aikins S. K.. New York: Springer. 219–232. doi: 10.1007/978-1-4614-1448-3_14

[ref14] Darley J. M. Latané B. (1968). Bystander intervention in emergencies: diffusion of responsibility. J. Pers. Soc. Psychol. 8, 377–383. doi: 10.1037/h0025589, PMID: 5645600

[ref15] De Vreese C. H. (2005). News framing. Doc. Des. 13, 51–62. doi: 10.1075/idjdd.13.1.06vre

[ref16] Dhanesh G. S. Rahman N. (2021). Visual communication and public relations: visual frame building strategies in war and conflict stories. Public Relat. Rev. 47:102003. doi: 10.1016/j.pubrev.2020.102003

[ref17] Dodonov R. (2022). Transformation of commemorative practices in Ukrainian historical discourse. Skhid 3, 5–14. doi: 10.21847/1728-9343.2022.3(1).253628

[ref18] Duncombe C. (2017). Twitter and transformative diplomacy: social media and Iran–US relations. Int. Aff. 93, 545–562. doi: 10.1093/ia/iix048

[ref19] Duncombe C. (2019a). Digital diplomacy: emotion and identity in the public realm. Hague J. Dipl. 14, 102–116. doi: 10.1163/1871191X-14101016

[ref20] Duncombe C. (2019b). The politics of twitter: emotions and the power of social media. Int. Political Sociol. 13, 409–429. doi: 10.1093/ips/olz013

[ref21] Dyczok M. Chung Y. (2022). Zelens′ kyi uses his communication skills as a weapon of war. Can. Slavonic Papers 64, 146–161. doi: 10.1080/00085006.2022.2106699

[ref22] Entman R. M. (1993). Framing: toward clarification of a fractured paradigm. J. Commun. 43, 51–58. doi: 10.1111/j.1460-2466.1993.tb01304.x

[ref23] Fang X. Zhong X. (2022). Algorithmic cognitive warfare: paradigm shift of public opinion warfare in the context of Russia-Ukraine conflict. Media Bserver 460, 5–l5. doi: 10.19480/j.cnki.cmgc.2022.04.003

[ref24] Fesun G. Fedirchyk T. Oliynyk M. (2019). Socio-psychological aspects of forming leader's image. Int. J. Soc. Educ. Innovat. 6, 29–36. Available at: https://www.journals.aseiacademic.org/index.php/ijsei/article/view/130

[ref25] Fu Z. Yan H. Zhong H. (2023). The operational mechanism, modular coordination, and practical insights of social media diplomacy. Southeast Commun., 20, 80–84. doi: 10.13556/j.cnki.dncb.cn35-1274/j.2023.08.031

[ref26] Gainous J. Wagner K. M. (2014). *Tweeting to power: The social media revolution in American politics*. New York: Oxford University Press.

[ref27] Gamson W. A. Modigliani A. (1989). Media discourse and public opinion on nuclear power: a constructionist approach. Am. J. Sociol. 95, 1–37. doi: 10.1086/229213

[ref28] Gardner W. L. Avolio B. J. (1998). The charismatic relationship: a dramaturgical perspective. Acad. Manag. Rev. 23, 32–58. doi: 10.2307/259098

[ref29] Gartzke E. (2013). The myth of cyberwar: bringing war in cyberspace back down to earth. Int. Secur. 38, 41–73. doi: 10.1162/ISEC_a_00136

[ref30] Goffman E. (1956). The presentation of self in everyday life. Doubleday.

[ref31] Goffman E. (1974). Frame analysis: An essay on the organization of experience. Cambridge, MA: Harvard University Press.

[ref32] Griffin M. (2004). Picturing America’s ‘war on terrorism’ in Afghanistan and Iraq. Journalism 5, 381–402. doi: 10.1177/1464884904044201

[ref33] Griffin E. (2006). A first look at communication theory: McGraw-Hill.

[ref34] Guo L. (2012). The application of social network analysis in agenda setting research: a methodological exploration. J. Broadcast. Electron. Media 56, 616–631. doi: 10.1080/08838151.2012.732148

[ref35] Guo L. McCombs M. (2011) Network agenda setting: A third level of media effects. In: Annual Conference of the International Communication Association, Boston, MA.

[ref36] Güran M. S. Özarslan H. (2022). Framing theory in the age of social media. Selçuk Üniversitesi Sosyal Bilimler Enstitüsü Dergisi 48, 446–457. doi: 10.52642/susbed.1142562

[ref37] Hamilton F. Bean C. J. (2005). The importance of context, beliefs and values in leadership development. Bus. Ethics Eur. Rev. 14, 336–347. doi: 10.1111/j.1467-8608.2005.00415.x

[ref38] Hanneman R. A. Riddle M. (2005). Introduction to social network methods. Riverside, CA: University of California.

[ref39] Hendrick C. Costantini A. F. McGarry J. McBride K. (1973). Attention decrement, temporal variation, and the primacy effect in impression formation. Mem. Cogn. 1, 193–195. doi: 10.3758/BF03198093, PMID: 24214516

[ref56] Hordecki B. Nosova B. (2023) Volodymyr Zelensky’s presidential rhetoric as a strategic resource. Przegląd Strategiczny. 13, 237–250.

[ref40] Hu Z. Li S. (2024). Digital presence: reconstructing the communication paradigm of visual images in the age of social media. Media Bserver. 41, 64–71. doi: 10.19480/j.cnki.cmgc.2024.02.003

[ref41] Iasiello E. (2015). Are cyber weapons effective military tools? Military Strategic Affairs 7, 23–40. Available at: https://www.inss.org.il/he/wp-content/uploads/sites/2/systemfiles/2_Iasiello.pdf

[ref42] Jensen M. J. Anstead N. (2014). Campaigns and social media communications: a look at digital campaigning in the 2010 U.K. general election. Inte. Democr. Glob. Perspect. 31, 57–81. doi: 10.1007/978-3-319-04352-4_5

[ref43] Jin X. Ning Z. (2023). New characteristics and trends in international political communication reflected by the Russia-Ukraine conflict. Jo. Shanxi Norm. Univ. (Soc. Sci. Ed.) 31:44-52+104. doi: 10.16207/j.cnki.1001-5957.2023.01.012

[ref44] Kalinchuk O. (2023) Emotional appeal in presidential rhetoric during the war period: case of president Zelenskyy during 2022 Russian invasion of ukraine. Vilniaus universitetas.

[ref45] Karlsen R. (2015). Followers are opinion leaders: the role of people in the flow of political communication on and beyond social networking sites. Eur. J. Commun. 30, 301–318. doi: 10.1177/0267323115577305

[ref46] Kaur G. Kaur A. Khurana M. Damaševičius R. (2024). Sentiment polarity analysis of love letters: evaluation of TextBlob, Vader, flair, and hugging face transformer. ComSIS 21, 1411–1433. doi: 10.2298/CSIS240328040K

[ref47] Kreis R. (2017). The “tweet politics” of president trump. J. Lang. Polit. 16, 607–618. doi: 10.1075/jlp.17032.kre

[ref48] Lakoff G. Johnson M. (2008). Metaphors we live by. Chicago: University of Chicago Press.

[ref49] Lambert M. J. (2022). Shaping the message: an analysis of US National Security Strategy Message Framing by the government and Media: Robert Morris University.

[ref50] Laruelle M. (2022). The Russian radical right and the war in Ukraine: a zealous Avant Garde, Dissident Voices, and Their Audience. The Mershon Center. Ohio State University.

[ref51] Lecheler S. De Vreese C. H. (2012). News framing and public opinion. J. Mass Commun. Q. 89, 185–204. doi: 10.1177/1077699011430064

[ref52] Liu Y. He J. (2021). “Why are you running away from social media?” analysis of the factors influencing social media fatigue: an empirical data study based on Chinese youth. Front. Psychol. 12:674641. doi: 10.3389/fpsyg.2021.674641, PMID: 34621208 PMC8490631

[ref53] Manor I. Crilley R. (2018). Visually framing the Gaza war of 2014: the Israel ministry of foreign affairs on twitter. Media War Conflict 11, 369–391. doi: 10.1177/1750635218780564

[ref54] Maskun M. Rum A. R. (2021). Cyber warfare: national security in dealing with changing method of war. Kanun Jurnal Ilmu Hukum 23, 477–490. doi: 10.24815/kanun.v23i3.22371

[ref55] Newton J. H. (1998). The burden of visual truth: the role of photojournalism in mediating reality. Vis. Commun. Q. 5, 4–9. doi: 10.1080/15551399809363390

[ref57] Park M. J. Kang D. Rho J. J. Lee D. H. (2016). Policy role of social media in developing public trust: twitter communication with government leaders. Public Manag. Rev. 18, 1265–1288. doi: 10.1080/14719037.2015.1066418

[ref58] Patrikarakos D. (2017). War in 140 characters: how social media is reshaping conflict in the twenty-first century. New York: Basic Books.

[ref59] Paul S. Das S. (2023). Investigating information dissemination and citizen engagement through government social media during the COVID-19 crisis. Online Inf. Rev. 47, 316–332. doi: 10.1108/OIR-06-2021-0307

[ref60] Perreault G. Paul N. (2018). An image of refugees through the social media lens: a narrative framing analysis of the humans of New York series ‘Syrian Americans’. J. Appl. Journal. Media Stud. 7, 79–102. doi: 10.1386/ajms.7.1.79_1

[ref61] Price V. Tewksbury D. (1996). Measuring the third-person effect of news: the impact of question order, contrast and knowledge. Int. J. Public Opin. Res. 8, 120–141. doi: 10.1093/ijpor/8.2.120

[ref62] Renaud K. Attatfa A. Craig T. (2022). “Positioning diplomacy within a strategic response to the cyber conflict threat” In: International Workshop on Socio-Technical Aspects in Security (Cham: Springer International Publishing). 13176, 131–152.

[ref63] Semetko H. A. Valkenburg P. M. (2000). Framing European politics: a content analysis of press and television news. J. Commun. 50, 93–109. doi: 10.1111/j.1460-2466.2000.tb02843.x

[ref64] Shi A. Pan J. (2023). Performance on social media platforms: a new path for political communication in the smart media era. Youth Journalist. 83, 95–99. doi: 10.15997/j.cnki.qnjz.2023.01.013

[ref65] Shi A. Tong T. (2020). Digital public diplomacy under the COVID-19 epidemic: challenges and innovation. Int. Commun. 5, 24–27.

[ref66] Shi A. Zhang Y. (2020). Digital public diplomacy: evolution of concepts. Youth Journalist 7, 78–81. doi: 10.15997/j.cnki.qnjz.2020.07.032

[ref67] Shonhe L. Jain P. (2017) Information dissemination in the 21st century: the use of mobile technologies. Information and Knowledge for Competitiveness, 425–447.

[ref68] Singer P. W. Friedman A. (2014). Cybersecurity: what everyone needs to know. New York: Oxford University Press.

[ref69] Soedarsono D. K. Mohamad B. Akanmu M. Putri I.-P. (2020). Political leaders and followers’ attitudes: twitter as a tool for political communication. J. Crit. Rev. 7, 1245–1252. doi: 10.5373/JARDCS/V12I2/S20201359

[ref70] Son J. Lee H. K. Jin S. Lee J. (2019). Content features of tweets for effective communication during disasters: a media synchronicity theory perspective. Int. J. Inf. Manag. 45, 56–68. doi: 10.1016/j.ijinfomgt.2018.10.012

[ref71] Specia M. (2022). Like a weapon”: Ukraini-ans use social media to stir resistance. The New York Times:25.

[ref72] Steblyna N. (2020). Selling insecurity via twitter: Ukrainian President’s posts and modern political discourse. Przegląd Strategiczny 10, 317–331. doi: 10.14746/ps.2020.1.19

[ref73] Sullivan J. (2019). The primacy effect in impression formation: some replications and extensions. Soc. Psychol. Personal. Sci. 10, 432–439. doi: 10.1177/1948550618771003

[ref74] Swartz D. (2003). “Pierre Bourdieu’s political sociology and governance perspectives” in Governance as social and political communication, 140–158.

[ref75] Szostek J. (2020). What happens to public diplomacy during information war? Critical reflections on the conceptual framing of international communication. Int. J. Commun. 14:21. Available at: https://ijoc.org/index.php/ijoc/article/view/13439/3092

[ref76] Tabansky L. (2011). Basic concepts in cyber warfare. Milit. Strat. Affairs 3, 75–92. Available at: https://www.inss.org.il/wp-content/uploads/sites/2/systemfiles/(FILE)1308129610.pdf

[ref77] Tasente T. (2020). Twitter discourse analysis of US president Donald Trump. Tech. Soc. Sci. J. 2:67. doi: 10.47577/tssj.v2i1.49

[ref78] Taverner A. (2010). “The military use of soft power–information campaigns: the challenge of application, their audiences and effects” in Soft power and US foreign policy. eds. Parmer I. Cox M. (London: Routledge), 149–163.

[ref79] Terry K. Yang F. Yao Q. Liu C. (2023). The role of social media in public health crises caused by infectious disease: a scoping review. BMJ Glob. Health 8:e013515. doi: 10.1136/bmjgh-2023-013515, PMID: 38154810 PMC10759087

[ref80] Uali B. Gabdulina B. Askeyeva G. (2023). The role of social media in sustainable development and strengthening the image of a political leader: Kazakhstan experience. Rivista Di Studi Sulla Sostenibilita 13, 91–108. doi: 10.3280/RISS2023-001005

[ref81] Van Gorp B. van der Goot M. J. (2012). Sustainable food and agriculture: Stakeholder's frames. Commun. Cult. Crit. 5, 127–148. doi: 10.1111/j.1753-9137.2012.01135.x

[ref82] Yang J. Counts S. (2010) Predicting the speed, scale, and range of information diffusion in twitter. In: Proceedings of the International AAAI Conference on Web and Social Media, 4, 355–358.

[ref83] Yavetz G. Aharony N. (2021). Social media for government information dissemination: content, characteristics and civic engagement. Aslib J. Inf. Manag. 73, 473–496. doi: 10.1108/AJIM-07-2020-0201

[ref84] Yu Y. (2018). The impact of social media on leadership authority from the perspective of media context theory: a case study of U.S. President Donald Trump. J. News Res. 9, 69–70.

[ref85] Zhan D. (2018). An examination of narrative types in news visualization production: Analysis Based on the Visual Reports of Sina and Xinhua News Websites. J. Univ. 38, 9–17+147.

